# The ANK repeats of Notch-4/Int3 activate NF-κB canonical pathway in the absence of *Rbpj* and causes mammary tumorigenesis

**DOI:** 10.1038/s41598-017-13989-7

**Published:** 2017-10-20

**Authors:** Ahmed Raafat, Sharon Bargo, David McCurdy, Robert Callahan

**Affiliations:** Basic Research Laboratory, National Cancer Institute, National Institutes of Health, Bethesda, MD 20814 USA

## Abstract

Transgenic mice expressing the Notch-4 intracellular domain (designated Int3) in the mammary gland have two phenotypes exhibited with 100% penetrance: arrest of mammary alveolar/lobular development and mammary tumorigenesis. Notch-4 signaling is mediated primarily through the interaction of Int3 with the transcription repressor/activator Rbpj. Interestingly, WAP-Int3/Rbpj knockout mice have normal mammary gland development but still developed mammary tumors with a slightly longer latency than the WAP-Int3 mice. Thus, Notch-induced mammary tumor development is Rbpj-independent. Here, we show that Int3 activates NF-κB in HC11 cells in absence of Rbpj through an association with the IKK signalosome. Int3 induced the canonical NF-κB activity and P50 phosphorylation in HC11 cells without altering the NF-κB2 pathway. The minimal domain within the Int3 protein required to activate NF-κB consists of the CDC10/Ankyrin (ANK) repeats domain. Treatment of WAP-Int3 tumor bearing mice with an IKK inhibitor resulted in tumor regression. In a soft agar assay, treatment of HC11-Int3 cells with P50-siRNA caused a significant decrease in colony formation. In addition, Wap-Int3/P50 knockout mice did not develop mammary tumors. This data indicates that the activation of NF-κB canonical signaling by Notch-4/Int3 is ANK repeats dependent, Rbpj-independent, and is mediated by IKK activation and P50 phosphorylation causing mammary tumorigenesis.

## Introduction

Notch plays an oncogenic role in human breast cancer development. The increase of intracellular Notch and enhanced Notch signaling were observed in a variety of human breast carcinomas^1–4^. Also, Lobular and ductal carcinomas expressed higher levels of Notch-1 and Notch-4 than the normal breast tissue^5–7^. The aberrant Notch signaling has been involved in the EMT induction during which cancer cells acquire an invasive phenotype^6–8^. These observations, together with the report that high expression of Jagged1 and/or Notch-1 are associated with poor clinical outcomes^1^ has resulted in an intense interest in exploring Notch signaling as a therapeutic target for breast cancer treatment^5,9,10^. Activation of Notch-1 or Notch-4 signaling pathways promoted the malignant behaviors of cells; whereas deactivation of these signals led to the reversal of the malignant cellular behavior^11–13^. A total of 8 gene fusion rearrangements involving either Notch-1 or Notch-2 were discovered in a panel of 89 breast cancer cell lines and tumors. These gene fusions lead to the expression of the Notch intracellular domain^14^. The aberrant activation of Notch-4 signaling pathway has been proven to be associated with the development and progression of breast cancers. High Notch-4 levels were detected in 81% of infiltrating ductal carcinomas (IDCs) and 93% of infiltrating lobular carcinomas (ILCs)^10^ none of the normal breast tissues expressed high levels of Notch-4.

Notch-4 was originally identified as a common integration site (CIS) for the mouse mammary tumor virus (MMTV) in mouse mammary tumors^15^. The viral genome integrates within Notch-4 causing the aberrant expression of the portion of the Notch-4 gene encoding the intracellular domain (ICD designated Int3). The expression of Int3 corresponds to a gain of function mutation. Expression of Int3 confers on HC11 normal mouse mammary epithelial cells the capability for anchorage independent growth in soft agar^16^. Int3 expressed as a transgene under the control of either MMTV LTR or the whey acidic protein (WAP) promoter in transgenic mice has two consequences that occur with 100% penetrance: the inability to lactate due to no mammary lobular/alveolar development and the development of mammary tumors^17,18^. Recently we have shown that these two phenotypes are a consequence of at least two different components of the Notch-4/Int3 signaling pathway^13^. Rbpj is a transcription repressor/activator that is a major partner in Notch signaling during development^19,20^. In the absence of Notch-ICD it acts as a transcription repressor. In the presence of Notch-ICD it binds to the Notch-ICD and becomes a transcription activator^20^. When we genetically crossed WAP-Int3 mice with Rbpj^−/−^ knockout mice we developed a WAP-Int3**/**Rbpj ^−/−^ mouse strain that exhibited normal mammary gland development which could lactate. However, these mice still developed mammary tumors^13^. This is consistent with WAP-Int3 mammary tumor development being independent of Notch-4/Int3-Rbpj signaling.

The specific roles and the underlying mechanisms of Notch-4 signaling pathway on the malignant behavior of breast cancer are poorly understood. To date, the Rbpj-independent mechanism whereby Notch-4 promotes cell transformation is not clear. The NF-κB family is among the targets of activated Notch^21–25^. NF-κB is a family of transcription factors that play a critical role in regulating cell survival, inflammation, differentiation, and proliferation. The NF-κB subunits exist in inactive form in the cytoplasm due to binding to inhibitory proteins of the IκB family (IκBs). Upon stimulation, an IKB kinase (IKK) complex is activated by IKK kinases^26,27^. This activated IKK complex phosphorylates IκBs, leading to the degradation of IκB and release of NF-κB, enabling NF-κB subunits to translocate to the nucleus^26^.

Several reports documented the cross talk between Notch signaling and NF-κB. Song *et al*.^22^, showed that Notch-1 associates with IKKα and regulates IKK activity in cervical cancer cells. Also, Notch-1 regulates NF-κB activity in hematopoietic progenitor cells^28^. Notch augments NF-κB activity by facilitating its nuclear retention^24^. In Notch-3 transgenic mice, NF-κB is constitutively active due to Notch-3 interaction with IKKα^29^. The activation of NF-κB signaling pathway by Notch signaling^11,22,25^ suggests that Notch tumorigenic signaling pathway may be mediated through the activation of NF-κB signaling pathway. Transcripts of NF-κB -regulated genes were found elevated in tumor initiating cells^30,31^ and breast tumors, as compared to normal tissue^31,32^. Several studies revealed high levels of constitutive NF-κB activity in many breast cancer cells^30,33–35^. Other studies showed NF-κB2 overexpression in breast and colon carcinomas^35–37^. NF-κB1 (P50) is overexpressed in colon, lung and breast cancers^35,37^. Therefore it has been considered a potential anti-cancer target^38,39^. In the current study we analyzed the Notch-4/Int3-Rbpj-independent tumorigenic signaling pathway. The data shows that Notch-4/Int3 activation of NF-κB canonical signaling complex is Rbpj-independent. This activation is dependent on the CDC10/Ankyrin repeats in Int3 which induces mammary tumors but not blockage of mammary gland development.

## Materials and Methods

### Mice, experiment design and preparation of tissue for morphology and histology analysis

WAP-Int3 female mice used in this study are maintained in our colony and have been described previously^18^. To delete P50 in mammary epithelial cells we crossed the WAP-Int3 mice with the P50^−/−^ mice (Jacksons lab, Bar Harbor, ME, USA). Tail DNA genotyping was performed by PCR for P50^−/−^ knockout using the following primers: forward, 5′- GCAAACCTGGGAATACTTCATGTGACTAAG-3′; and reverse 5′- ATAGGCAAGGTCAG AATGCACCAGAAGTCC-3′. DNA was amplified (94 C for 30 sec., 68 C for 30 sec., 72 C for 30 sec; 35 cycles, 72 C for 2 min) with a product size of 192 bp. To treat WAP-Int3-tumor-bearing mice with IMD-0354^39^, primary mammary tumors were palpated weekly. Tumor weight was determined as described previously^12^. When mammary tumors reached 400 mg, mice were euthanized and mammary tumors were collected as viable tissue. To reduce inter-tumor variations, nulliparous FVB/N female mice from our colony were used at 10 weeks of age and the inguinal mammary glands of these FVB/N mice served as the transplantation site of the primary WAP-Int3 viable tumor tissue. Viable tissue from each WAP-Int3 mammary tumor was placed in the inguinal mammary gland of two separate FVB/N mice. Once tumors reached the desired weight, one mouse received IMD-0354 and the matching control mouse received saline. Alzet mini osmotic pumps (Model 2001, pumping rate 1 µl/h, Durect Corp., Cupertino, CA, USA), implanted subcutaneously on the dorsal side of the mouse, were used to deliver a subcutaneous dose of IMD-0354 (5, 10 or 20 mg/mouse/week) or saline (control). Mammary whole mounts were prepared from the fourth abdominal gland, as previously described^13^. Mice were kept under standard laboratory conditions per the guidelines of the National Cancer Institute. All methods were performed in accordance with the NIH Guide for the Care and Use of Laboratory Animals. The National Cancer Institute (NCI) Animal Care and Use Committee approved all experimental procedures.

### Cell culture and gene silencing

HC11^40,41^ and HC11-Int3 mouse mammary epithelial cells were grown in RPMI medium. The HC11-Int3 cell line was generated as described previously^42^. Gene silencing using siRNA was done as previously described^43^ Briefly, knockdown of proteins was achieved by the treatment of cells with siRNA (siGenome SMART pool, Dharmacon/Thermo Scientific Lafayette, CO) according to the manufactures protocol. The siRNA transient transfections were done with a final concentration of 50 nM in six-well plate or scaled up 10 cm-plate formats.

### Evaluation of NF-κB activity

The Active Motif (Carlsbad, CA, USA) TransAM^TM^ NF-κB family ELISA kit was used to facilitate the study of the NF-κB family transcription factors (P50, P52, P65 and Rel-B) in extracts of HC11 and HC11-Int3 cells treated with Int3-siRNA or Rbpj-siRNA according to the manufacturer’s specifications. NF-κB activity was measured in nuclear protein extracts. The assay was performed according to the manufacturer’s protocol as described previously^22^. Samples were analyzed using a microplate absorbance reader Bio-rad680 (Bio-Rad, Hercules, CA, USA).

### Construction of Int3 deletion mutants

The murine NICD-4 cDNA corresponding to a truncated Notch4/Int3 cDNA (residues 4382–6043) has been described^15,17^. An oligonucleotide encoding hemagglutinin (HA) tag was added to the 3′ end of NICD-4/Int3 cDNA. The HA-tagged NICD-4/Int3, RAM 23, CDC10/ANK, RAM-ANK and PB-PEST expression vectors were generated through PCR using *Pfu* polymerase from StrataGene (La Jolla, CA, USA) and cloned into the eukaryotic expression vector pcDNA3 as described previously^44^. The nucleotide sequence of all NICD-4/Int3 deletion plasmids have been determined and their sequences verified. The Int3 PCR deletion products were cloned into the *Nhe*I and *Xho*I site of the pcDNA3.1(−) (Invitrogen, Carlsbad, CA, USA). The protein encoded by the PCR product was synthesized using TNT Coupled Transcription/Translation System (Promega, Madison, WI, USA) according to the manufacturer’s specifications and as described previously^45^. The synthesized protein was analyzed by electrophoresis on a 10% SDS-polyacrylamide gel (SDS-PAGE) and autoradiography. To measure the ability of the Int3 deletion mutants to activate NF-κB P50 signaling in HC11 cells containing an NF-κB reporter gene (Qiagen, Valencia, CA), cells were transiently transfected with expression vectors containing the Int3 deletions.

### Colony formation in soft agar and luciferase assay

The soft agar assay utilizes an agar medium to assess the transformability of cells in an anchorage-independent manner. Soft agar assay was done as described previously^12^. For luciferase Assays, cells were grown in DMEM medium containing 10% fetal bovine Serum. Transfection and luciferase assays were conducted as previously described^12^.

### Cell migration and invasion assays

Cell invasion and migration across a basement membrane matrix were evaluated using a commercially available 12-well plate cell invasion/migration assay kit (Chemicon International, Temecula, CA, USA) and following the manufacturer’s instructions^46^. Briefly, 2 × 10^5^ cells were seeded into individual invasion chambers, and subsequently placed in 12-well plates containing serum (10% FBS) culture medium in the lower chamber and incubated for 24 and 48 h. Non-invading cells were carefully wiped off the upper surface of the filters with a swab. Cells that invaded and migrated through the matrix-containing membrane and reached the lower surface of the invasion chamber were stained with crystal violet and counted in at least three different high power fields using a light microscope.

### Immunoblotting and Immunoprecipitation

Cells were harvested with trypsin-EDTA (1x) (Gibco BRL, San Francisco, CA, USA) and collected as a pellet by centrifugation at 4 °C. Cells were lysed and processed for Western blot analysis as described previously^43^. After blotting, membranes were probed overnight with primary antibodies to HA (ab1818, Abcam, Cambridge, MA, USA), Notch-4 (07–189, Millipore, Billerica, MA, USA), IKKα (2682, Cell Signaling Technology, Danvers, MA, USA), IKKβ (2370, Cell Signaling Technology, MA, USA), NF-kB/P50 (Santa Cruz SC114, Sacramento, CA, USA) or P50 blocking peptide (Santa Cruz SC114P, Sacramento, CA, USA). Membranes were washed and incubated with horseradish the proper peroxidase (HRP)-conjugated secondary antibody (1:5000; Amersham Biosciences, Piscataway, NJ, USA). ECL reagent (Amersham Biosciences, Piscataway, NJ, USA) was used for detection. For Immunoprecipitation**, c**ells were harvested in RIPA buffer supplemented with the protease inhibitor cocktail set 1 (Calbiochem, San Diego, CA, USA). Total protein concentration was determined with the BCA Protein Assay Kit (Pierce Biotechnology, Rockford, IL, USA). Total protein lysate (500 µg) was immunoprecipitated, blotted and detected as described previously^43^.

### Statistics

Quantitative values are represented as the mean of at least three experiments. All *in vivo* experiments were repeated at least three times, and at least five mice were used in each experiment. The statistical significance of the difference between groups was determined by the Wilcoxon rank sum test. Comparisons resulting in *P*-values ≤ 0.01 were considered statistically significant and identified in the figures with an asterisk (^*^) or (**).

## Results

**Figure 1:**
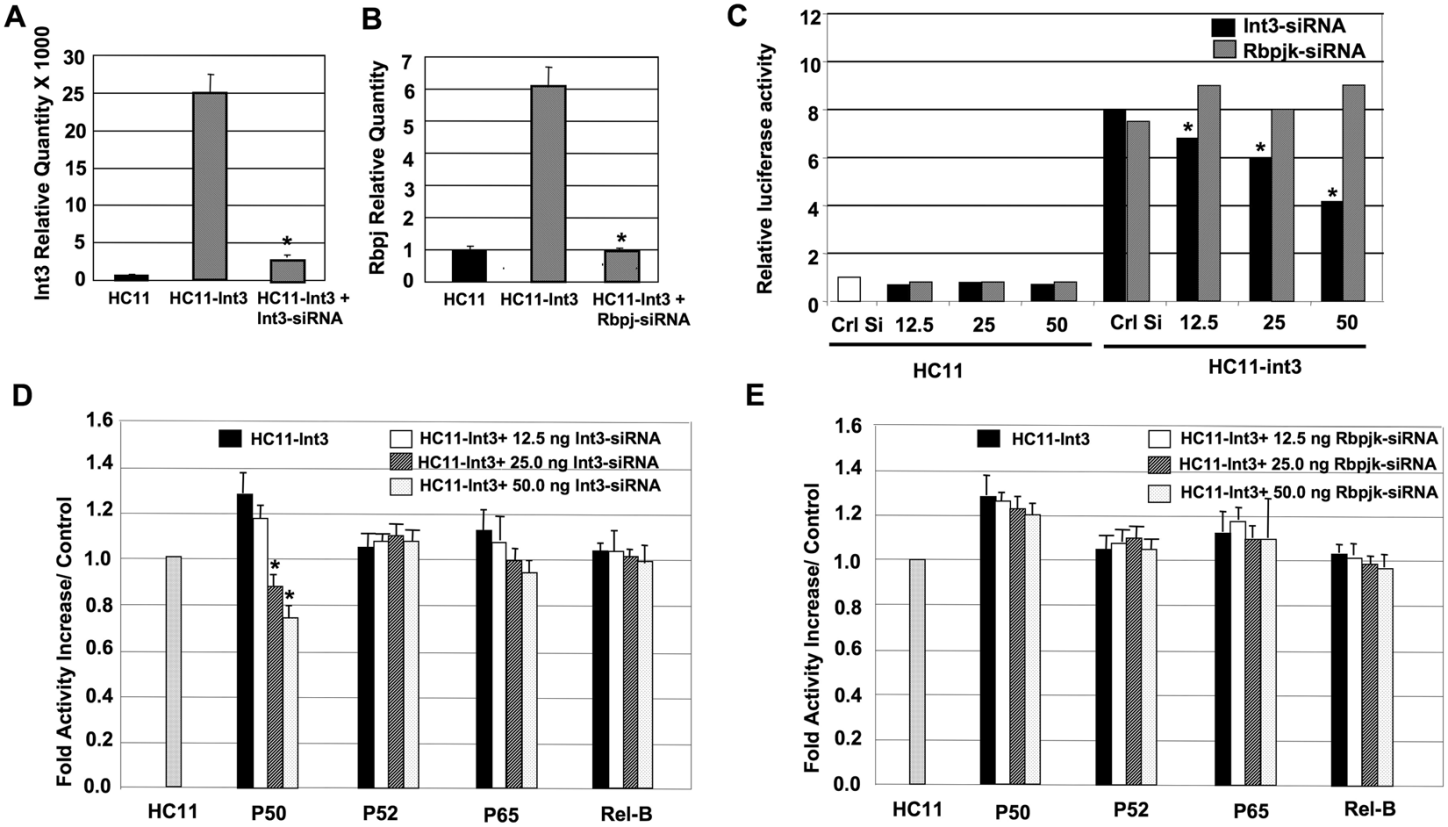
Int3 and NF-κB cross-talk. Int3 (**A**) and Rbpj (**B**) siRNA treatment of HC11-Int3 cells caused a significant reduction in Int3 and Rbpj expression. Levels of Rbpj and Int3 in HCll and HC11-Int3 mammary epithelial cells was conducted as described in the Materials and Methods. (**C**) Int3 modulates NF-κB signaling in Rbpj-independent manner. Luciferase assays in HC11 and HC11-Int3 cells expressing the NF-κB-luc promoter reporter and the control Renilla luciferase reporter were either mock transfected (Crl Si) or transfected with increasing doses of Int3-siRNA or Rbpj-siRNA. NF-κB-promoter activity is significantly reduced when Int3-siRNA increased. *P ≤ 0.01, compared to HC11-Int3-NF-κB-Luc. (**D**) Int3 binding to NF-κB transcription factors. HC11-Int3 cells were cultured and treated with increasing doses of Int3-siRNA (12.5, 25 and 50 ng) as described in the Materials and Methods. (**E**) Binding of Int3 to NF-κB promoter in absence of Rbpj. HC11-Int3 cells were cultured and treated with increasing doses of Rbpj-siRNA (12.5, 25 and 50 ng) as described in the Materials and Methods. For panel D and E; nuclear proteins from each treatment were extracted and incubated with plate bound NF-κB-DNA. The effects of siRNA treatment on the levels of DNA binding to NF-κB subunits were assessed by the TransAM-NF-κB Family ELISA-Kit. Results are reported as the mean ± SEM (n = 3) and the data are expressed considering 1.0 as the relative binding capacity of untreated HC11 cells ^*^P < 0.01 compared to HC11-Int3. In all panels, each bar represents the mean ± SEM of a minimum of a duplicate of three independent experiments for each experimental group.

Involvement of NF-κB signaling in Notch induced tumorigenesis has been suggested from studies that demonstrate its activation in Notch-1 tumors^11,22^. To investigate whether Notch-4/Int3 signaling activates NF-κB and whether this activation is Rbpj-dependent or -independent, we used Notch-4-siRNA (Fig. 1A) and Rbpj-siRNA (Fig. 1B) to inhibit Notch-4 and Rbpj-RNA expression in HC11-Int3 cells after incubating these cells with TNF-α. TNF-α was added to stimulate the NF-κB pathway, however the used dose (5 ng) did not induce NF-κB in absence of Int3 (Fig. 1A). The respective siRNA did knock down the levels of the target mRNA (Fig. 1A,B). We investigated the effects of Notch4/Int3 on NF-κB activation/signaling by analyzing the effects of Int3 on activation of NF-κB promoter driven-reporter gene in HC11-Int3 cells in presence of increasing doses of Int3-siRNA (Fig. 1C). Int3 significantly induced NF-κB promoter-driven luciferase. This activity is suppressed in presence of Int3-siRNA in a dose dependent manner. Previous data showed that Int3 mammary tumorigenesis is Rbpj-independent^13^. To investigate if NF-κB -reporter activation is Rbpj-dependant/independent, HC11-Int3 cells were transfected with the NF-κB -reporter in presence of increasing concentrations of Rbpj-siRNA (Fig. 1C). Rbpj-siRNA treatment did not affect the NF-κB -driven luciferase activity, indicating that Notch4/Int3 activation of NF-κB is Rbpj-independent.

To verify that Int3 activation of NF-κB is Rbpj-independent and to investigate the activation of NF-κB canonical (P50 and RelA/P65) and non-canonical (P52 and RelB) pathway transcription factors by Int3; we quantified NF-κB transcription factors (P50, P52, P65 and Rel-B) activation when Int3 is knocked down with Int3-siRNA using TransAm NF-κB kit. Binding of P50 to NF-κB binding site decreased significantly in absence of Int3 in a dose dependent manner (Fig. 1D). Similarly, but to a lesser extent, P65 binding to the NF-κB binding site decreased (Fig. 1D). P52 and Rel-B binding to the NFκB binding site do not appear to be affected by Int3 expression. In addition, we performed experiments where HC11-Int3 cells are treated with increasing doses of Rbpj-siRNA. Reduction of Rbpj expression in HC11-Int3 cells did not affect the binding of P50 or P65 to the NF-κB binding site (Fig. 1E) indicating that the Int3/NFκB-P50 or P65 interaction/activation is Rbpj-independent. These results demonstrate that modulation of Int3 levels affects NF-κB activation, in Rbpj-independent fashion. Since P65 binding to the NF-κB binding site was not significant we have focused on Int3 interaction with the NF-κB1/P50.

**Figure 2:**
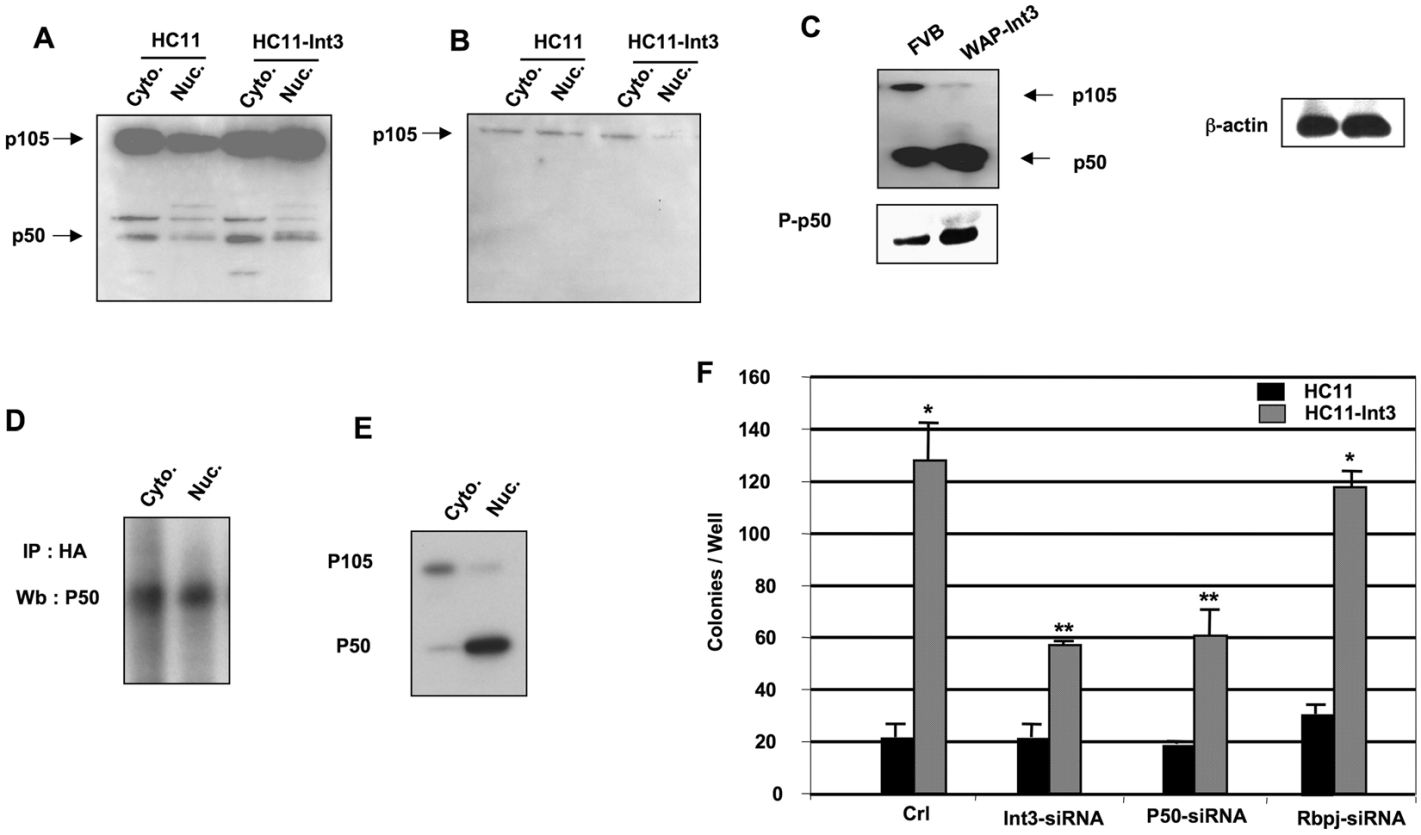
Physical binding of Int3 to NF-κB-P50 and NF-κB-P65. (**A**) and (**B**) P50 antibody control panels. (**A**) Western blot analysis of cytoplasmic and nuclear protein extracts of HC-11 and HC-11- Int3 cells. P50 AB detected P105 and P50 proteins. (**B**) Western blot analysis of cytoplasmic and nuclear protein extracts of HC11 and HC11-Int3. Protein extracts treated with P50 peptide. P50 antibody did not detect P50 in presence of the competing P50 peptide. (**C**) Western blot analysis of the levels of P50, phosphorylated P50 in total protein extracts of mammary glands from late pregnant FVB/N and WAP-Int3 mice. The extract loading control was evaluated by reacting the blot with β-actin antibody. (**D**) Interaction of Int3 and P50. Cytoplasmic and nuclear protein extracts were immunoprecipitated with antibody to the HA tag expressed in HC11-Int3-HA cells. The immunoprecipitates and cell extracts were electrophoretically separated on a polyacrylamide gel and transferred to a nitrocellulose filter. The filters were reacted with antibody to NF-κB-P50, indicating binding of Int3 to P50.Western blot analysis of P50 (**E**) levels in cytoplasmic and nuclear extracts of HC11-Int3-HA cells. Int3 induces P105 processing in HC11-Int3-HA cells. (**F**) The effect of siRNA treatment on soft agar growth of HC11 and HC11-Int3 cells. HC11 and HC11-Int3 cells were treated with siRNA for Rbpj, NF-κB P50 and Int3. In each assay 15,000 cells were plated in soft agar to measure their capability for anchorage independent growth. *P < 0.01 compared to HC11, **P < 0.01 compared to HC11-Int3. Each bar represents the mean ± SEM of a minimum of a duplicate of three independent experiments for each experimental group.

Although various homo- and heterodimeric combinations possibly function in the NF-κB/Rel family, the heterodimers of NF-κB1/P50 and RelA/P65 in the canonical pathway as well as the heterodimers of NF-κB2/P52 and RelB in the non-canonical pathway are considered to be predominant components of the respective pathways^26,27,47^. To examine the effects of Notch-4-Int3 on the status of P105/P50 we employed P50 antibody. To test the specificity of the P50 antibody Western blots analysis of HC11 and HC11-Int3 nuclear and cytoplasmic extracts were reacted with the antibody. Major bands were detected at 105 KD and 50 KD (Fig. 2A). Reactivity with these bands was blocked by the P50 blocking peptide (Fig. 2B). We have further characterized this interaction in the mammary glands of late pregnant FVB and WAP-Int3 mice, the P105 precursor of P50 is more abundant in the FVB mammary gland whereas in the WAP-Int3 gland total p50 and phosphorylated P50 are more abundant than in the FVB gland (Fig. 2C), indicating activation of P105 by Int3. This data is in agreement with previous studies^11,21,23,48,49^.

To obtain evidence that Int3 and P50 physically interact, an immune-precipitate (IP) and Western blot (wb) analysis was performed on TNF-α treated HC11-Int3-HA cell. cytoplasmic and nuclear protein extracts (Fig. 2D). Cytoplasmic and nuclear protein extracts were subjected to immunoprecipitation with HA antibody. Western blot of the immunoprecipitates were probed with the anti-P50 (Fig. 2D) antibody. Immunostaining analysis revealed a predominant Int3 binding to P50 in both cytoplasmic and nuclear extracts. Western blot analysis of HC11-Int3-HA cytoplasmic and nuclear extracts showed that the predominant species in the cytoplasmic fraction was P105 precursor and in the nuclear fraction the predominant species was P50 (Fig. 2E), indicating activation of P105 in presence of Int3.

The capability for anchorage-independent growth by tissue culture cells in soft agar is accepted as a measure of their tumor-inducing potential. We have shown previously^13,16^ that the HC11 mouse mammary epithelial cells cannot grow in soft agar, whereas HC11-Int3 cells do have this capability^13^. To investigate the effect of NF-κB canonical pathway knockout on HC11-Int3 soft agar growth capability, we treated HC11 and HC11-Int3 cells with, P50-siRNA or Int3-siRNA (Fig. 2F). If the Int3/P50 complex is required to activate NF-κB pathway and confer the capability for Int3 anchorage-independent growth of HC11-Int3 cells, then blocking the expression of P50 should block growth in soft agar of these cells. As shown in Fig. 2F, soft agar growth significantly decreased in absence of P50 compared to HC11-Int3. Knocking down Rbpjk with siRNA has little or no effect on the ability of HC11-Int3 cells to form colonies in soft agar, indicating that Int3 colony formation is Rbpj-independent^13^. These results are compatible with the conclusion that the ability of HC11-Int3 cells for anchorage-independent growth in soft agar is independent of an Int3/Rbpj signaling pathway^13^ and is NF-κB dependent. As a positive control we used Int3-siRNA to block Int3 expression in the HC11-Int3 cells and as expected colony formation was knocked down (Fig. 2F).

**Figure 3:**
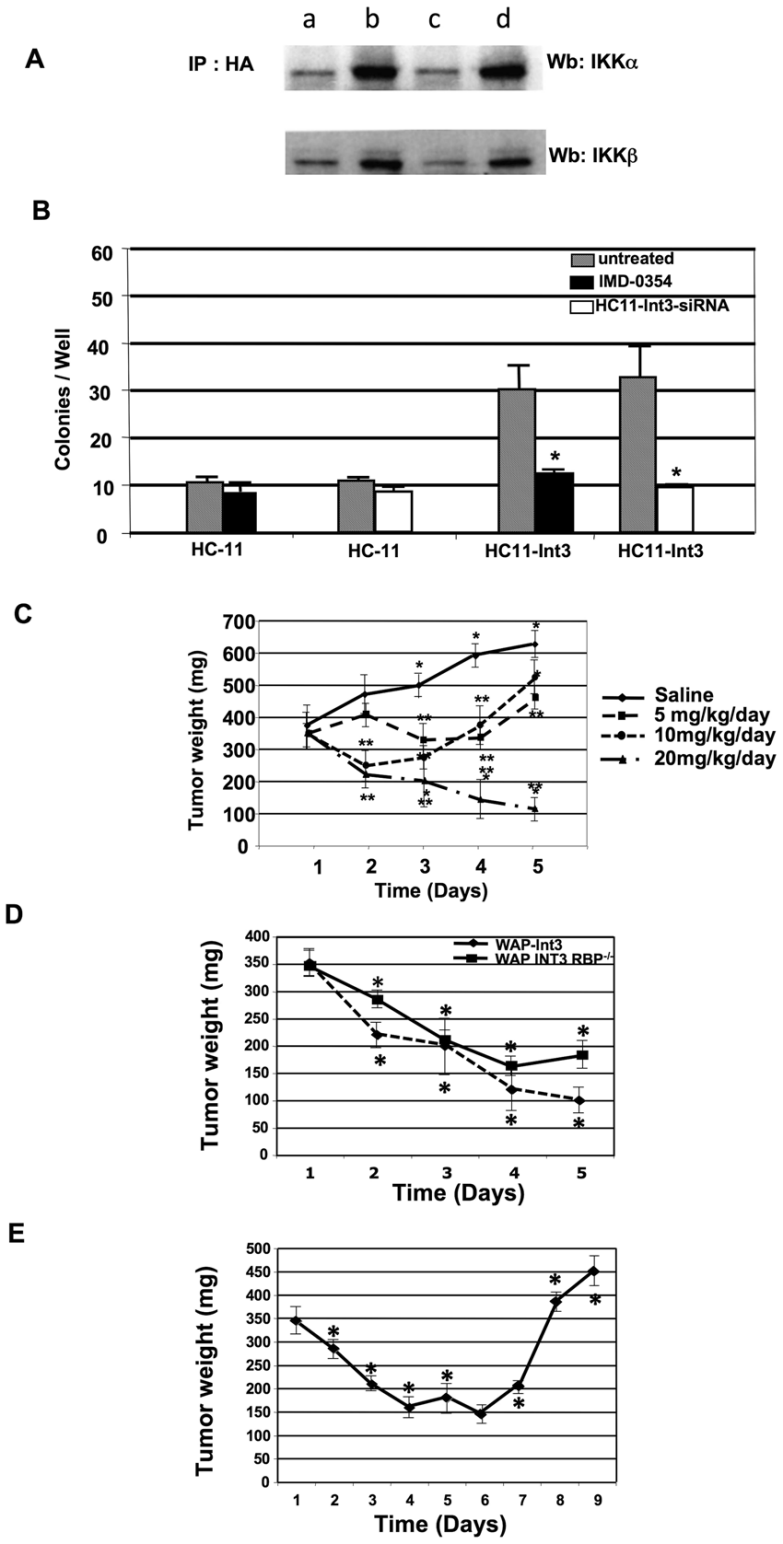
The interaction between Int3 and IKKα and IKKβ. (**A**) Co-immuno-precipitation of Int3 with IKKα and IKKβ. Protein extract were prepared from untreated (a) HC11, (b) HC11-Int3 cells and HC11 Int3 cells treated with either (c) Int3-siRNA or (d) Rbpj-siRNA (d) were immunoprecipitated with HA antibody. The immunoprecipitates were analyzed by Western blot analysis using IKKα or IKKβ antibody as described in the Materials and Methods. Decreased levels of IKKα and IKKβ were bound to Int3 in HC11-Int3 cells treated with Int3 siRNA as compared to untreated cell or cells treated with Rbpj siRNA. (**B**) Int3 transformation ability is IKKβ dependent. Blocking of IKKβ with inhibitor IMD-0354 (5 μM) in HC11-Int3 caused a significant reduction in colony formation in soft agar assay. The reduction is comparable to blocking of Int3 expression by siRNA. (**C**) Treatment of WAP-Int3 tumor bearing mice with the IKKβ inhibitor IMD-0354. A dose response to 5, 10 and 20 mg/kg/day of IMD-0354. Note that the tumors regressed in the tumor bearing mice receiving 20 mg/kg/day. *Significantly different than day 1, **significantly different from saline control. n = 8 mice/time point. (**D**) Effect of IMD-0354 treatment on WAP-Int3 Rbpj^−/−^ mammary tumors. WAP-Int3 and WAP-Int3 Rbpj^−/−^ tumor bearing mice were treated for 5 days with 20 mg/kg/day. *Significantly different from day 1. Note that the tumor regression in both strains was virtually identical. n = 8 mice/time point (**E**) When treatment was terminated (day 5) the tumors grew back. n = 8 mice/time point.

Others have shown that Notch-1 binds to the IKKα signalosome^22^. Therefore, to better understand the cross-talk between Noth-4-Int3 and NF-κB we investigated the binding of Int3 to IKKα and IKKβ. Protein extracts were prepared from HC11-HA and HC11-Int3-HA cells treated with either Int3-siRNA or Rbpj- siRNA. Immunoprecipitates were prepared using an antibody to the HA tag and electrophoretically separated on a polyacrylamide gel followed by blotting on a filter. The filters were reacted with an antibody to either IKKα or IKKβ. As shown in Fig. 3A (lane c) when HC11-Int3-HA cells were treated with Int3-siRNA the level of reactive IKKα and IKKβ was decreased to similar levels as seen in HC11 extracts (Fig. 3A, lane a). Whereas in extracts from cells treated with Rbpj-siRNA (Fig. 3A, lane d) the levels of bound IKKα and IKKβ were similar to that found in immunoprecipitates from untreated HC11-Int3-HA cells (Fig. 3A, lane b). We conclude that both IKKα and IKKβ interact with Int3 independent of Rbpj.

IKK signalosome phosphorylation of IκB leads to its proteosomal degradation and the translocation of P50/P65 hetrodimers to the nucleus. IMD-0354 is a specific IκB kinase-β (IKKβ) inhibitor^39^ that blocks NF-κB nuclear translocation. To ascertain whether members of the NF-κB family are involved in Notch-4/Int3-induced mammary tumorigenesis, first we treated HC11, HC11-Int3 with IMD-0354 in the presence and absence of Int3-siRNA (Fig. 3B). Blocking of NF-κB nuclear translocation by IMD-0354 significantly reduced Notch-4/Int3 transformation capacity to levels similar to those observed when HC11-Int3 cells were treated with Int3-siRNA. To further investigate the role of NF-κB in Int3 tumorigenesis, we undertook a dose-response study to determine the effects of IMD-0354 on WAP-Int3 tumor-bearing mice. Mice treated with a 20 mg/week dose showed significant reduction in the tumor weight compared to the saline treated WAP-Int3 tumor bearing mice (Fig. 3C). Therefore, a dose of 20 mg/week was used in all subsequent experiments. Histological analysis of hematoxylin and eosin (H&E)-stained liver and kidney sections from IMD-0354-treated mice did not show any evident morphological alterations such as necrosis or inflammatory changes due to toxicity (data not shown). These results demonstrate that WAP-Int3 mammary tumors are sensitive to IMD-0354 and further provide a strong rationale that the activation of one or more of the NF-κB target transcription proteins, in the context of Int3 signaling, likely contributes to tumor growth. To determine if this tumor regression by IKKβ inhibition is affected by Rbpj status, we treated WAP-Int3/Rbpj^−/−^ tumor-bearing mice with 20 mg/week IMD-0354 for one week and monitored tumor weight (TW) daily. At the end of the treatment period, TW was significantly reduced (Fig. 3D) in all mice. When the pumps were depleted of IMD-0354 after day 6 (Fig. 3E), there was a resumption of tumor growth, suggesting that continuous inhibition of IMD-0354 targets (IKKβ) is needed to effectively inhibit tumor growth. These results demonstrate that Notch4/Int3 mammary tumors are sensitive to IKKβ/NF-κB inhibition and further provide a strong evidence that the Notch-4/Int3 mammary tumorigenesis is NF-κB dependent and Rbpj independent.

**Figure 4:**
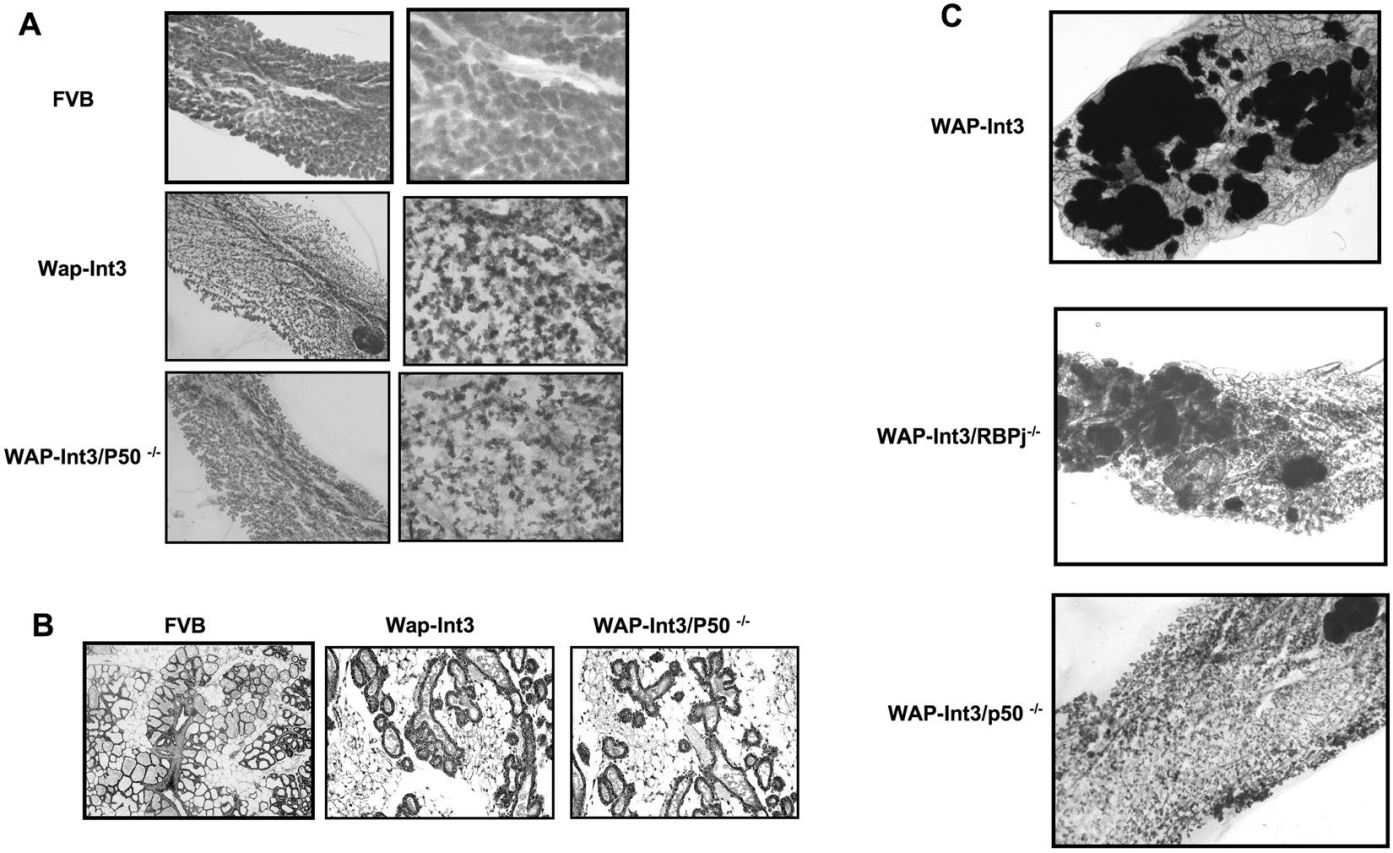
Effect of NFκB P50 deletion in WAP-Int3 mice on mammary gland development and mammary tumorigenesis. (**A**) Whole mount of number 4 inguinal mammary gland at day 1 of first lactation of FVB, WAP-Int3 and WAP-Int3/P50^−/−^. (**B**) Histological analysis showed impaired side-branching and alveolar development in the WAP-Int3 and WAP-Int3/P50^−/−^ compared to FVB. Note there is no discernible difference between the WAP-Int3 and WAP-Int3/P50^−/−^ mammary glands. (**C**) *In vivo* effects of P50 deletion on Int3 mammary tumorigenesis. Multiple subclinical tumors can be observed in whole mounts of WAP-Int3 and WAP-Int3 Rbpj^−/−^ mice at their second lactation. This is not the case in mammary glands from WAP-Int3 P50^−/−^. A total of at least 8 mice were used for each experiment.

To further dissect these results, we have examined the effect of loss of NF-kB-P50 on WAP-Int3 mammary tumor development by developing a WAP-Int3/NF-κB-P50^−/−^ mouse strain. It should be noted that the parental NF-kB-P50^−/−^ female mice have no mammary gland developmental phenotype^50^. Like WAP-Int3 mice, WAP-Int3/NF-κB-P50^−/−^ mice cannot lactate due to an arrest in mammary lobular/alveolar development (Fig. 4A,B). In whole mounts of Wap-Int3 and Wap-Int3/Rbpj^−/−^ mammary glands one can observe multiple independent tumor-like growths^13^ (Fig. 4C). Significantly, WAP-Int3/NF-κB-P50^−/−^ mice do not develop mammary tumors even after 4–5 pregnancies (Table 1). These results provide further evidence that Notch-4/Int3 mammary tumor development is NF-κB1 dependent and Rbpj independent.

**Table 1:** Lactation and tumor development in WAP-Int3 and WAP-Int3/P50^−/−^ mice mammary glands.

Strain	Pregnancy	Lactation	Tumor
WAP-Int3	2	No	80%
WAP-Int3/P50 −/−	4:5	No	0%

In fact, even after 4–5 pregnancies WAP-Int3/P50^−/−^ do not develop tumors (n = 8) whereas after 2 pregnancies 80% of WAP-Int3 parous females have developed mammary tumors (n = 8).

**Figure 5:**
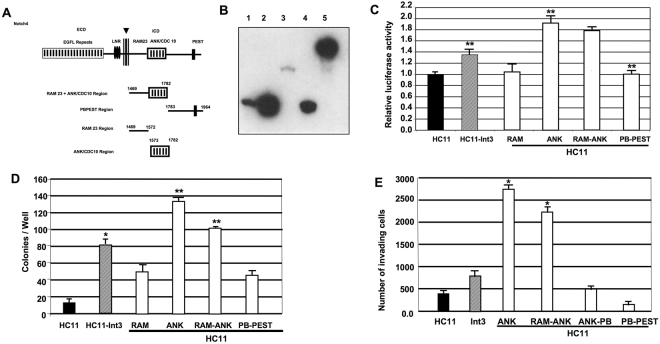
Characterization of the minimal regions of Notch4/Int3 required for intermolecular activation of NF-κB. (**A**) To map the region(s) of NICD4/Int3 that mediate interaction with NF-κB, we generated a series of Int3 deletion constructs. The regions of Int3 that remain in each deletion mutant is indicated. The numbers above the maps indicate an amino acid residue at indicated boundaries. (**B**) *In vitro* translation products of the Int3 deletions. RAM (lane 1), ANK (lane 2), RAM-ANK (lane 3), PB-PEST (lane 4) and full length Int3 (lane 5). (**C**) NF-κB reporter assay of HC11, HC11-Int3, HC11-RAM, HC11-ANK, HC11-RAM-ANK, HC11-PB-PEST cells. The ANK repeats region of Int3 caused the highest activation of NF-κB reporter. **P < 0.01 compared HC11 cells. (**D**) HC11 cells were transfected with expression vectors expressing the Int3, RAM, ANK, RAM-ANK, and PB-PEST deletion mutants compared to HC11 and HC11-Int3 cells for their ability to confer on HC11 cells the capability for anchorage independent in soft agar. (**E**) HC11 cells expressing Int3, RAM, ANK, RAM-ANK and PB-PEST deletion mutants compared to HC11 and HC11-Int3 cells in an invasion assay (see Materials and Methods). In panel (C–E), each bar represents the mean ± SEM of a minimum of a duplicate of three independent experiments for each experimental group.

To further define the minimal region of Int3 required for activating NF-κB, we have created a series of Int3-HA tagged deletion mutants that encode the RAM, ANK/CDC10, RAM-ANK and PB-PEST regions. Shown in Fig. 5A is a map of the regions of Int3 remaining in the deletion mutants. In Fig. 5B is shown the *in vitro* translated gene products expressed by the different deletion mutants. We have assayed HC11 cells transfected with expression vectors expressing these Int3 deletion mutants for NF-κB reporter activity (Fig. 5C). The HC11-RAM and HC11-PB-PEST cells were unable to activate the NF-κB reporter gene whereas HC11-ANK and HC11-RAM-ANK cells were capable of activating the reporter gene (Fig. 5C). In addition, HC11-ANK and HC11-RAM-ANK cells were capable of forming colonies in soft agar assay (Fig. 5D). Similarly, HC11-ANK and HC11-RAM-ANK cells showed significant increase in the number of invading cells in an invasion assay (Fig. 5E). These results are consistent with the Int3-ANK region being responsible for the activation of NF-κB.

## Discussion

The WAP-Int3 transgenic mice exhibit two phenotypes with 100% penetrance^18^. One phenotype is the blockage of mammary gland lobulo-alveolar development resulting in an inability of WAP-Int3 females to lactate. We have previously shown that this phenotype is dependent on the interaction of the Notch4-ICD**/**Int3 with the transcription repressor/activator Rbpj^13^. The other phenotype is the development of mammary tumors in all females that is independent of Int3/Rbpj signaling^13^. The aim of the present study is to investigate and dissect the Notch/Rbpj independent signaling pathway. A limited microarray analysis of HC11-Int3 RNAs revealed high steady-state levels of IL1, IL6, IL-8, CXCL1 and CXCL2, as compared to control HC11 cells (our unpublished data). These genes are targets for the NF-κB transcription factors^51,52^. NF-κB pathways are important for normal mammary gland development as well as for breast cancer tumorigenesis and cancer stem cell biology^31,53^. In response to multiple stimuli, IKB kinase (IKK) complex is activated by IKK kinases^26^. Activated IKK complex consisting of two catalytic subunits (IKKα and IKKβ) and an IKKγ regulatory subunit in turn phosphorylates IκBs at two conserved serine residues in the N-terminal domain, leading to its proteasome mediated degradation. As a result, NF-κB translocates to the nucleus and induce a variety of genes encoding matrix metalloproteinases, inflammatory and chemotactic cytokines, and antiapoptotic proteins^26,47,54^. Several animal model studies linked NF-κB canonical and non-canonical pathway to inflammation and cancer. The canonical pathway is activated by IκB kinase (IKK α/β/γ complex), activation of RelA/P50 and the non-canonical pathway activated by NF-κB -inducing kinase (NIK) and IKKα, activation of RelB/ P52 pathways^26^.

A reporter assay showed that activation of NF-κB by Int3 is Rbpj-independent (Fig. 1C). The activation of NF-κB by Notch-4/Int3 indicates that in addition to a role for Notch in proliferation, differentiation, and survival of tumor cells^44,55^, Notch-4/Int3 may also have critical functions in the immune response, inflammation, viral infection, and apoptosis through control of NF-κB-mediated gene expression. Int3 enhanced P50, and to a lesser extent P65 (Rel-A), binding to the NF-κB DNA binding site but did not affect the binding of P52 or Rel-B to this site. High levels of P50 in presence of Notch-4/Int3, can alter the ratio of p50/p50 versus p65/p50 NF-κB dimers, thereby affecting the selectivity of the canonical NF-κB-dependent transcription. In addition, we have shown that Int3 binding to P50 is Rbpj-independent (Fig. 1E). Furthermore, knocking down P50 prevents anchorage independent growth by HC11-Int3 cells in soft agar (Fig. 2E) regardless of Rbpj presence. This suggests that the ability of Int3 to confer on HC11 cells the capability of anchorage independent growth is a consequence of Int3 activating the canonical NF-κB signaling pathway. In contrast, it has been reported that Notch-1-ICD interacts with the P50 subunit of NF-κB^56^, but not P65^22^, and blocks the formation of P50/ P65 heterodimers to NF-κB binding sites, thereby interfering with NF-κB-induced transcriptional regulation. Song *et al*.^22^ have shown that Notch-1 activation of NF-κB in CaSki cells is Rbpj dependent. The crosstalk between NF-κB and Notch pathways is quite complex and depends on the exact pathophysiological context. Thus, the difference between what we have found and what has been reported in the literature may reflect either the cellular context the assays were performed in or the divergence between the Notch-1 and Notch-4-ICDs.

Others have shown that Notch-ICD Interacts with NF-κB-P50 and the IKK signalosome^22^. Therefore, our observation that Int3 binds to IKKα/IKKβ raised the possibility that inhibition of IKK could inhibit the ability of Int3 to induce tumor growth. Therefore, we treated mice bearing WAP-Int3 tumors with the IκB kinase-β (IKKβ) inhibitor IMD-0354^39^ that blocks NF-κB nuclear translocation^39,57^. In each case using 20 mg/kg/day the tumors totally regressed and the effect of the drug was independent of presence or absence of Rbpj. When the drug was removed the tumor grew back. These results, along with the inhibition of HC11-Int3 cells anchorage independent growth in soft agar by P50-siRNA, are consistent with the hypothesis that it is the canonical NF-κB signaling that Int3 activates during mammary tumorigenesis. To test this hypothesis, we have developed a WAP-Int3**/**NF-κB-P50^−/−^ mouse strain. It should be noted that the parental NF-κB-P50^−/−^ mouse strain has no mammary gland developmental phenotype^50^. Like WAP-Int3 mice, WAP-Int3**/**NF-κB-P50^−/−^ can’t lactate due to the absence of lobulo-alveolar development that we have previously shown to be a consequence of Int3/Rbpj signaling^13^. 80% of WAP-Int3 females develop tumors after the second pregnancy^13^, however, WAP-Int3**/**NF-κB-P50^−/−^ mice did not develop mammary tumors after 4–5 pregnancies (Table 1). These results are consistent with the conclusion that the consequence of the Int3/NF-κB1-P50 interaction is mouse mammary tumorigenesis. Interestingly, we were also able to obtain two WAP-Int3**/**NF-κB-P50^−/−^/Rbpj^−/−^ females which could lactate and did not develop mammary tumors i.e. no phenotype (data not shown). Again, consistent with the conclusion that Int3/Rbpj signaling affects mammary gland development and Int3/NF-κB canonical signaling results in mammary tumorigenesis.

Previously MacKenzie *et al*. demonstrated that the Notch-4 induced inhibition of endothelial sprouting requires the ANK repeats in the ICD-for Rbpj dependent and independent signaling^58^. Subsequently we showed that the Tacc3 protein binds specifically to the Notch-4 ANK repeats and inhibits Notch-4/Rbpj signaling^59^. Having established that Int3 binds to P50 we have genetically dissected, using deletion analysis, the region(s) of Int3 that are responsible for binding to P50. We found that Int3-ANK and RAM-ANK, but not RAM or PB-PEST, activates the expression of NF-κB reporter gene in HC11 cells. We conclude that only the ANK region is required for activation of NF-κB, since RAM by itself did not induce NF-κB reporter activity.

Dumont E., *et al*., showed that the ANK region of Notch1-ICD is required for neoplastic transformation in collaboration with Adenovirus E1A protein and is independent of transcription activation by Rbpj signaling^60^. In contrast Wang *et al*. reported that Notch-1 inhibits NF-κB mediated gene expression through a region N-terminal to the ANK repeat, namely, aa 1773–1881^56^. The differences between Notch-1 and Notch-4 regions that bind to NF-κB may reflect the divergence between the amino acid sequences of their ICDs.

Several reports have described the crosstalk between NF-κB and Notch. Most of these studies investigated interaction between Notch-1 and NF-κB. The reports have been conflicting, numerous studies indicated that Notch receptors activate NF-κB. This activation involves a group of components along the signal transduction pathway at multiple levels. For example, Notch activates NF-κB by increasing transcription expression of NF-κB subunits and NF-κB dependent genes^24,28,61^. Two different mechanisms have been elucidated in the physical interaction of Notch with NF-κB. First, physical interaction between Notch and P50/Rel-A^21,24^, trapping activated NF-κB in the nucleus. Second, interaction with the components of the signalosome. Recently, it has been shown that overexpression of Notch-1-ICD activates NF-κB by interacting with the IKKα/β signalosome and enhancing IKKα/β kinase activity in Caski cells^22^. Notch-3 can increase the association between IKKα and NIK, resulting in P50/P65 heterodimer nuclear entry and subsequent cell proliferation^62^. Notch signaling activation mediated by Jagged-1 is reported to induce IKK kinase activity in human keratinocytes^63^ and murine erythroleukemia^64^. In terms of Notch-4-Int3, it activates NF-κB by both mechanisms, interaction with P50 and interaction with the signalosome.

Several factors drive breast cancers to estrogen independence including down regulation of estrogen receptor (ER) expression, modulation of regulation of signal transduction pathways and ER mutations. It has been suggested that in ER independent breast tumors NF-κB expression contributes to a highly invasive and metastatic tumor that is chemotherapy resistant^64–66^. NF-κB was overexpressed in a majority of ER negative primary breast tumors and breast cancer cell lines compared to ER positive breast tumors and tumor cell lines^64,67,68^. Nakshatri *et al*.^64^ proposed that breast cancers that lack functional ER overexpress NF-κB-regulated genes. Also in the ERα positive cells, estrogen inhibited Notch signaling^5^. Indicating a cross-talk between ER, Notch and NF-κB signaling. This proposal is supported by our observations in the MMTV-Int3 transgenic mice, where endogenous ovarian estrogen secretion in the post-pubertal MMTV-Int3 mice blocked Int3 inhibitory effects on normal mammary development, namely ductal elongation^13,19^. Because of these considerations NF-κB has been regarded as a potential target for therapeutic intervention of ER negative breast cancer^65,69,70^. This inhibition can be achieved by blocking Notch signaling in the ER negative breast cancers.

**Figure 6:**
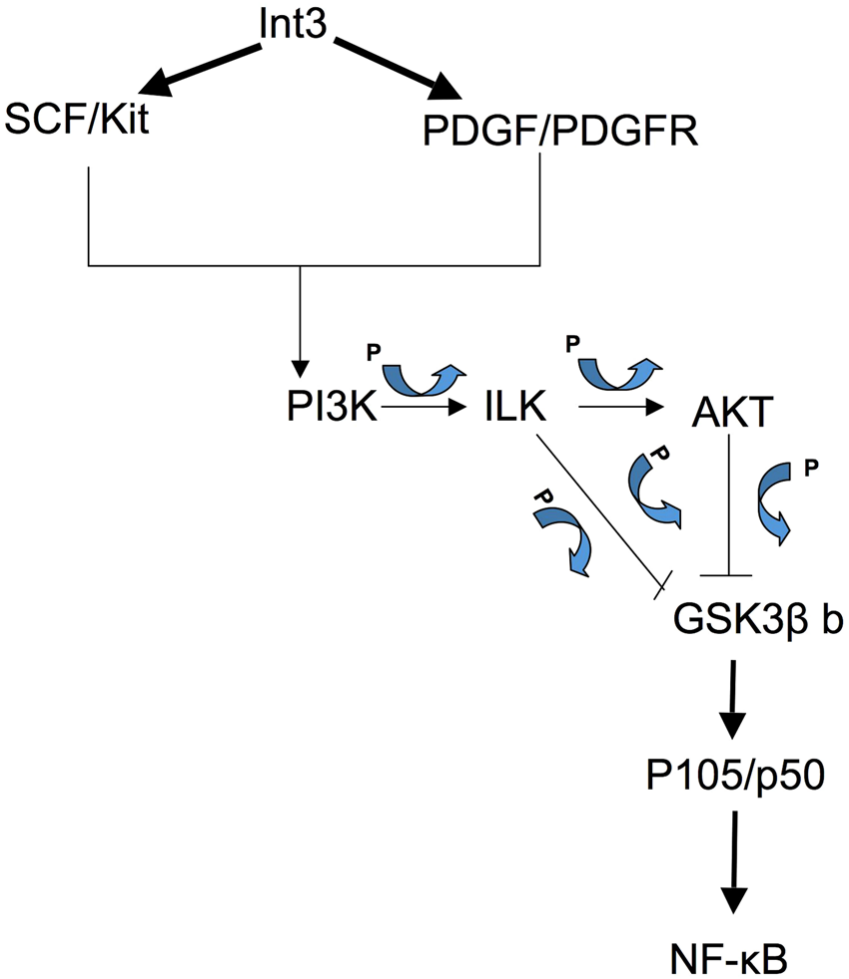
Proposed model. Int3 activates PI3K through PDGFR and cKit, leading to phosphorylation of ILK and AKT. This results in phosphorylation (deactivation) of GSK3β, and subsequent activation of canonical NF-κB pathway through induced processing of P105 to P50.

Inhibition of NF-κB enhanced the sensitivity of tumor cells to apoptosis induced by chemo drugs and radiation^71–73^. However, there is no specific NIK inhibitor for pharmaceutical use yet. Because of its important role in both canonical and non-canonical NF-κB pathways in the immunity and the severe phenotype of NIK-deficient mice^74^, inhibiting NIK may also cause severe side effects. A good alternative would be the agents that targets Notch signaling, such as tyrosine kinase inhibitors (Gleevec). Previously we showed that expression of activated Notch-4 -ICD (Int3) led to the up regulation of c-Kit and PDGFR-RNA expression^12^. Further, we showed that treating cells with the c-Kit/PDGR inhibitor Gleevec, leads to the activation of GSK3β^43^ (non-phosphorylated form of GSK3β). We have demonstrated that activated GSK3β was associated with the phosphorylation of Int3, its ubiquitination and subsequent degradation in the proteasomes^43^. Interestingly Foltz at al., have reported that Notch-1-ICD is also a target for GSK3β phosphorylation^75^, however in this case the phosphorylated Notch-1-ICD is stabilized from proteasome-mediated degradation. This is consistent with the differential modulation of some components of Notch signaling by GSK3β. Demarchi *et al*., have reported that GSK3β also regulates the stability of the NF-κB-P50 precursor P105^76^. In the absence of GSK3β activity or the GSK3β protein, P105 is constitutively processed to P50. Phosphorylation of P105 by GSK3β and subsequently by IKK leads to proteasome-mediated degradation of P105^77,78^. Using this model (Fig. 6) we propose that Notch-4-Int3 activates c-Kit and PDGFR, leading to activation of GSK3β and NF-κB signaling. In presence of Gleevec, GSK3β is dephosphorylated and as a result it will phosphorylate Int3 leading to its ubiquitination and degradation^43^. As a result, the ability of Int3 to induce P105 processing and NF-κB activation is diminished. Thus, Gleevec treatment of a Notch-4-ICD or Int3 positive mammary tumors/estrogen negative would be expected to be associated with proteasome-mediated degradation of P105, inhibition of NF-κB canonical pathway and subsequent remission of mammary tumor growth.
